# Evaluating the Effect of Random Multi‐Donor Pooling on the Nutritional Variability in Donor Human Milk Using Computer Modeling

**DOI:** 10.1111/mcn.70158

**Published:** 2026-01-08

**Authors:** R. Mitchell Smith, Scott Richter, Esther F. Iwayemi, Kimberly Mansen, Kiersten Israel‐Ballard, Daniela Hampel, Setareh Shahab‐Ferdows, Lindsay H. Allen, Lars Bode, Maryanne T. Perrin

**Affiliations:** ^1^ Department of Mathematics and Statistics University of North Carolina Greensboro Greensboro North Carolina USA; ^2^ Department of Pediatrics, Larsson‐Rosenquist Foundation Mother‐Milk‐Infant Center of Research Excellence (MOMI CORE), and the Human Milk Institute (HMI) University of California, San Diego La Jolla California USA; ^3^ Department of Nutrition University of North Carolina Greensboro Greensboro North Carolina USA; ^4^ Integrated Maternal and Child Health and Development, PATH Seattle Washington USA; ^5^ Department of Nutrition, Institute for Global Nutrition University of California Davis California USA; ^6^ United States Department of Agriculture, Agriculture Research Service Western Human Nutrition Research Center Davis California USA

**Keywords:** donor milk, milk banking, minerals, nutrition, preterm, vitamins

## Abstract

Protein and fat concentrations in donor human milk (DHM) can vary twofold to threefold and are influenced by the number of unique donors per pool. The aim of this study was to broadly characterize how the number of donors (2–10) randomly combined into a pool during milk bank processing influenced the variability of macronutrients, vitamins, minerals, and bioactive factors in DHM. The minimum number of donors required for 80% of the pools to meet pre‐defined targets for true protein, fat, and disialyllacto‐N‐tetraose (DSLNT) was also evaluated. Monte Carlo simulation was used to create models that accounted for donor lifetime donation volume and milk bank production constraints. Variability in nutrients was quantified as a Nutrient Inequality Index (NII) which was computed as the ratio of the 90th percentile to the 10th percentile for each simulation. Random multi‐donor pooling of 2–10 donors produced lower variability in DHM macronutrients than most vitamins and minerals. A priori targets of 0.9 g/dL of true protein, 3.5 g/dL of fat, and 210 µg/L of DSLNT could not be achieved with any random pooling scenario. The NII for lactose stabilized at less than 1.1 when there were 3+ donors per pool, while the NII for fat and true protein stabilized at less than 1.3 when there were 5+ donors per pool. The NII exceeded 1.5, even at 10 donors per pool, for several micronutrients including zinc, copper, sodium, iron, biotin, riboflavin, B6, B12, and pantothenic acid.

## Summary

- Donor human milk (DHM) macronutrient composition has been reported to vary by twofold or higher, and there is a dearth of information on micronutrient composition.
- Emerging evidence suggests that random multi‐donor pooling may be an effective strategy for milk banks to reduce nutritional variations in DHM.
- Random multi‐donor pooling of 2–10 donors produced lower variability in lactose, fat, and true protein, than most vitamins and minerals.
- At 5 to 10 donors per pool, several micronutrients varied by 1.5 to 2.0‐fold including zinc, sodium, iron, copper, thiamin, B6, B12, biotin, and panthothenic acid.

## 1 Introduction

The World Health Organization (WHO) provides a strong recommendation for the use of mother's milk for preventative and promotive care of the small vulnerable newborn (WHO Recommendations for Care of the Preterm or Low‐Birth‐Weight Infant 2022). In the absence of mother's own milk, donor human milk (DHM) is recommended (Academy of Pediatrics Committee on Nutrition, Section on Breastfeeding, & Committee on Fetus and Newborn 2016; WHO Recommendations for Care of the Preterm or Low‐Birth‐Weight Infant 2022), based on growing evidence that DHM significantly reduces the risk of necrotizing enterocolitis (NEC) compared to formula feeding (Colaizy et al. 2024; Quigley et al. 2024).

There is limited information on the nutritional composition of DHM (Perrin et al. 2020), with emerging evidence that the degree of variability differs by nutrients. For example, in a study of 300 samples of DHM collected systematically from 20 milk banks within the Human Milk Banking Association of North America (HMBANA) network, protein varied twofold (0.7–1.4 g/dL), fat varied over threefold (1.9–6.1 g/dL), and sodium varied over 13‐fold (42–569 mg/L) (Friend and Perrin 2020; Perrin et al. 2022). Similar variations in milk macronutrients were described in a recent study of Australian milk bank donors (Walter et al. 2023). Multi‐donor pooling is a strategy that can be used by milk banks to reduce the variability in DHM nutrients. It involves combining donations from multiple approved donors to create a more consistent profile of nutrients and bioactive factors. Young et al. found that the variation of protein, insulin, and immunoglobulin A decreased with more donors in the pool. We found that sodium concentrations varied over 13.2‐fold in single donor pools, compared to 2.4‐fold variation in pools that were made using milk from 4 to 5 unique donors (Perrin et al. 2022). Most research on the impact of multi‐donor pooling has considered 2–5 donors per pool and has focused on macronutrients or bioactive factors (Friend and Perrin 2020; John et al. 2019; Tabasso et al. 2023; Young et al. 2019), leaving a dearth of information related to micronutrients and higher donor pools.

The aim of this study was to broadly characterize how the number of unique donors (2–10) randomly combined into a pool during milk bank processing influenced the variability of macronutrients, vitamins, minerals, and bioactive factors in DHM. We also sought to determine the minimum number of donors required per pool to meet pre‐defined clinical targets for true protein, fat, and the human milk oligosaccharide disialyllacto‐N‐tetraose (DSLNT). These nutrients were selected based on their potential importance for preterm infants related to growth, energy, and NEC protection (Amissah et al. 2020; Autran et al. 2017; Masi et al. 2021; Perrin et al. 2023).

## 2 Methods

We used Monte Carlo simulations using R software (*R: The R Project for Statistical Computing* n.d) to evaluate the impact of the number of donors (2–10) on the nutrient variability of pooled donor human milk. Simulation was selected as a methodology to allow us to study the impact of pooling beyond 2‐5 donors per pool, which is the current upper limit used by most milk banks (Perrin et al. 2025).

Each simulation created 1000 theoretical pools of donor milk by randomly selecting a predefined number of unique donors—from 2 through 10—to use in each pool. Cross‐sectional data from the Global Donor Human Milk study served as the sampling frame for the simulations. This dataset included nutrients and bioactive information about the milk of 400 approved milk bank donors in the United States (100 donors from 4 milk banks), as well as their Lifetime Donation Volume to the milk bank. The sample collection protocol for this study has previously been described, as has the distribution of all study variables (Perrin et al. 2023, 2025). Briefly, each sample reflected deidentified milk collected from a milk bank in the HMBANA network. Each sample was from a unique, approved donor and had been pooled across multiple collection days. This sampling lens reflects the way that milk donations are processed within a milk bank, ensuring that our findings are generalizable to DHM.

### 2.1 The Simulation

#### 2.1.1 Donors Selection

For each theoretical pool, the donors were randomly selected using a weighted selection process that ensured that donors with a greater Lifetime Donation Volume were more likely to be selected. The rationale for this decision is that donors who provide high volumes of milk will be used more frequently in the DHM production process than donors who provide small volumes of milk. Once a donor was selected for a specific pool, they could not be selected again for that pool, though they could be selected again for future pools. Thus, within an individual pool there was no duplication in donors, but an individual donor may have been selected for multiple pools within the 1000 simulated pools.

#### 2.1.2 Determining Volume Contributed by Each Donor to Each Simulated Pool

The rules for determining the volume contribution of each donor were developed to create theoretical pools that reflected the typical production pooling volumes reported by the milk banks in the Global Donor Human Milk study (ranging from 16 to 65 L) as well as typical minimum donation volumes required to become a milk bank donor (approximately 3 L). We sought to have an upper limit for the Total Pool Volume of approximately 40 L, with no lower limit, as milk banks sometimes create small specialty pools of DHM. Different simulation scenarios used different rules to stay within upper limit constraints for total pool volume, which differed by a predefined volume constant (VOL_const_). For 2–6 donor pools, VOL_const_ = 2.5 L was used. For 7 and 8 donor pools, VOL_const_ = 2 L was used, and for 9 and 10 donor pools, VOL_const_ = 1.75 L was used. Each donor's volume contribution for each simulated pool was determined as follows:

- If donor's Lifetime Donation Volume was less than VOL_const_, all the donor's milk was contributed to the pool.
- If donor's Lifetime Donation Volume was greater than VOL_const_ we randomly sampled a MULTIPLIER from the following distribution of 6 multipliers: 1.25, 1.5, 1.75, 2.0, 2.25, or 2.50. The multiplier was selected at the donor‐level, not the pool level (so within a pool, one donor may have a multiplier of 1.25, and another may have a multiplier of 2.5). This allowed the volume contribution per donor to fluctuate, which is reflective of milk banking practices.
- If MULTIPLIER x VOL_const_ was greater than the donor's Lifetime Donation Volume, then all the donor's milk was contributed; otherwise, the volume contributed was equal to MULTIPLIER x VOL_const_.

#### 2.1.3 Accounting for Pasteurization Effects

The milk samples collected for the Global Donor Human Milk study were raw milk (Perrin et al. 2025). Because milk typically undergoes pasteurization in a milk bank setting, our data set also included 50‐matched samples that had been Holder pasteurized to allow us to account for the effects of pasteurization. The distribution of the gain/loss for each nutrient during the pasteurization process was utilized to estimate the effect of pasteurization on each nutrient for each combined pool of the simulation. Using the 50 matched samples, the mean and standard deviation of the change due to pasteurization was computed for each nutrient. Then, for each simulated pool, the mean of each nutrient was adjusted by adding a randomly selected change value from the corresponding distribution of mean change during pasteurization to produce a simulated post‐pasteurization value for each nutrient in that pool. Changes after Holder pasteurization were limited (0%–15% loss) for nutrients, while the impact on bioactive factors like lactoferrin were extensive (> 80%). These results have been published elsewhere (Davis et al. 2025).

#### 2.1.4 Pool Nutrient Composition and the Impact of Pooling

The final pool composition of each nutrient was computed by weighting the nutrient composition of each individual donor selected for the pool by the volume that each donor contributed to the pool and adjusting for the expected impact of Holder pasteurization.

To characterize the impact of pooling on the variability of each nutrient, we computed a Nutrient Inequality Index (NII) as the ratio of the 90th percentile to the 10th percentile. The NII provides a single value that can be used across nutrients at different scales (micrograms to grams) and can be interpreted clinically as the expected fold‐difference of the middle 80% of the distribution for each nutrient. For example, true protein concentrations of 0.70 and 0.88 g/dL for the 10th percentile and 90th percentile respectively, would produce an NII of 1.26 (0.88/0.70), This can be interpreted as 1.26‐fold difference in true protein values in the core 80% of DHM.

### 2.2 Model Verification and Statistical Analysis

We verified that our models were operating as intended by monitoring the simulated pool volumes and the contribution of each donor to the simulated pools. Total pool volume was calculated by summing the volume contributions of each individual donor that was randomly selected for the pool. Our goal was to create pools no larger than 35–40 L based on feedback from our milk banking partners. To verify that our simulations were including more milk from donors that contributed higher lifetime donation volumes, we computed the Simulated Cumulative Donor Volume Contribution from each donor throughout the entire simulation of 1000 pools. Pearson correlation coefficients were used to describe the relationship between Simulated Cumulative Donor Volume Contribution and the Actual Lifetime Donation Volume.

To increase trustworthiness in our models, we compared simulated NII values with empirical NII values derived from DHM samples that had been collected systematically from 20 HMBANA milk banks and assessed for lactose [2‐donor (*n* = 20); 3‐donor (*n* = 20); 4 donor (*n* = 19); 5‐donor (*n* = 11); unpublished data] and sodium [2‐donor (*n* = 83); 3‐donor (*n* = 121); 4‐donor (*n* = 33); 5‐donor (*n* = 11)] (Perrin et al. 2022). Lactose was selected as an example of a less variable nutrient and sodium was selected as an example of a more variable nutrient. Our model made assumptions about inventory turns, as this was not known. We tested the sensitivity to this assumption by modeling both Fast Turns (inventory was immediately available for the next simulated pool) and Slow Turns (inventory was not replaced until 500 pools had been compiled, and then the milk bank was considered restocked, returning all donor volumes to full capacity to generate the next 500 pools).

To characterize the impact of donor pooling, Nutrient Inequality Indices for each nutrient were graphed by the number of donors per pool to visualize the influence of multi‐donor pooling. We also evaluated the 20th percentile of each pooling scenario to determine if at least 80% of the pools met the following a priori minimum nutrient targets of: (1) true protein ≥ 0.9 g/dL; (2) fat ≥ 3.5 g/dL; and (3) DSLNT ≥ 210 µg/L. The rationale for these targets has been described elsewhere (Perrin et al. 2023). This research was classified as non‐human subjects research by Institutional Review Board at the University of North Carolina Greensboro (protocol IRB‐FY21‐68). As part of the regular donor screen processes that occurred at the individual milk banks where the samples were obtained, donors consent that their milk donations may be used for research purposes.

## 3 Results

Complete nutrient and volume data were available for 386/400 (96.5%) of the subjects, due to some sample loss that occurred during transportation and analyses, which served as the final sampling frame for the simulation. Table 1 provides descriptive information on the nutrient composition of the sampling frame.

**Table 1:** Descriptive statistics for the nutrient composition of raw milk samples from approved US milk bank donors (*n* = 386) used as the simulation sampling frame.

Analyte	Mean (Std dev)	Range
Lactation Stage (weeks)	17.8 (14.7)	‐0.5 – 120.6
Lifetime Donation (L)	58.4 (80.5)	0.7–623.8
True Protein (g/dL)	0.8 (0.2)	0.4–1.8
Lactose (g/dL)	6.7 (0.4)	4.5–7.9
Fat (g/dL)	3.2 (0.7)	1.2–5.8
Lactoferrin (mg/dL)	342 (181)	93– 2324
IgA (mg/dL)	151 (36)	63–451
Total HMOs (g/dL)	0.5 (0.2)	0.1–12.4
DSLNT (mg/L)	107 (73)	19–538
Thiamine (µg/L)	140 (29)	32–269
Riboflavin (µg/L)	288 (181)	90–1637
Niacin (µg/L)	1521 (547)	511–4531
Pantothenic Acid (µg/L)	4427 (2961)	532–23,049
B6 (µg/L)	152 (82)	7–650
Biotin (µg/L)	35 (139)	0.6–1846
B12 (pmol/L)	378 (219)	76–1122
WS choline (mg/L)	92 (22)	27–177
Retinol (mg/L)	0.4 (0.2)	0.1–1.2
α‐tocopherol (mg/L)	4.0 (1.8)	1.2–15.5
γ ‐tocopherol (mg/L)	1.0 (0.4)	0.3–2.6
Calcium (mg/L)	291 (47)	168–445
Copper (µg/L)	260 (133)	52–1590
Iron (µg/L)	176 (95)	0–631
Magnesium (mg/L)	33 (6)	18–67
Phosphorus (mg/L)	147 (24)	93–226
Potassium (mg/L)	532 (75)	373–794
Selenium (µg/L)	10.0 (2.6)	5.2–26.6
Sodium (mg/L)	119 (59)	41–784
Zinc (µg/L)	1597 (1004)	226–5276

Abbreviations: DSLNT ‐ disialyllacto‐N‐tetraose; HMO – human milk oligosaccharides; WS – water‐soluble.

### 3.1 Summary of Simulated Pools and Sensitivity to Inventory Turns

**Figure 1:**
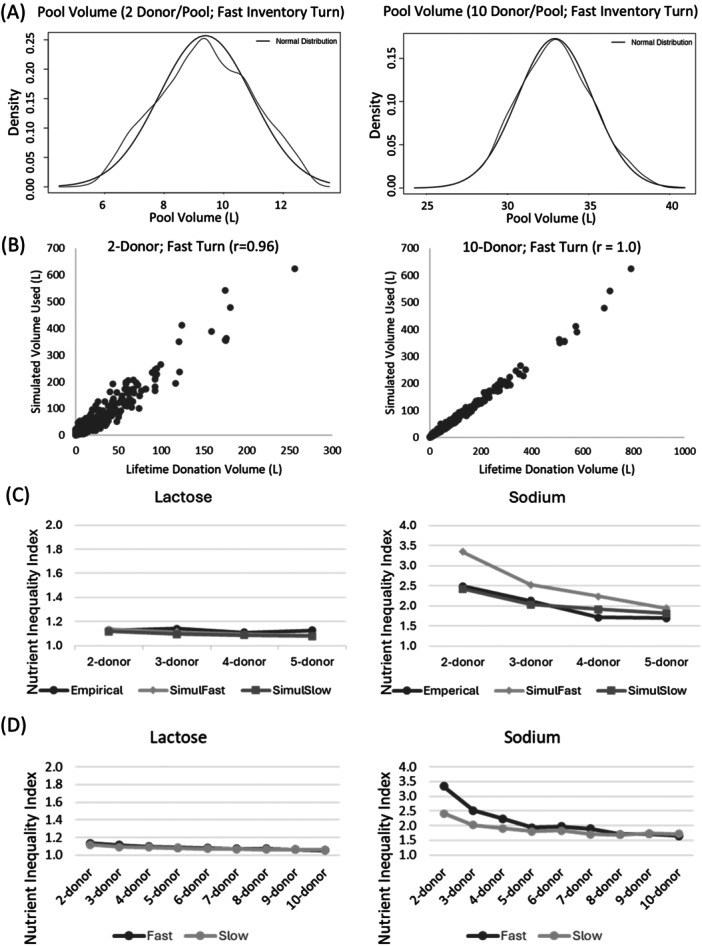
Verification of milk banking constraints using (A) kernel density plots for 2 and 10 donor pools at Fast Inventory Turns and (B) scatter plots of actual Lifetime Donation Volume to Simulated Cumulative Donor Volume at Slow and Fast Inventory Turns. (C) Comparison of models against empirical data for lactose (*n* = 70) and sodium (*n* = 248). (D) Sensitivity analysis of assumptions for inventory turnover (Slow vs. Fast) for lactose (example of a less variable nutrient) and sodium (example of a more variable nutrient). SimuFast – simulation using a fast inventory turnover assumption; SimuSlow – simulation using a slow inventory turnover assumption.

We first examined the characteristics of the simulated pools. Kernel density plots of pool volumes for 2 and 10 donor pools (Figure 1A) reflected the production capacity of the milk banks participating in this study (upper limit of 35–40 L per pool). The milk volumes used in the simulation were also strongly correlated with the lifetime donation volume of the donors (Figure 1B; *r* > 0.96 for all simulations) indicating that milk‐use in the model strongly reflected the actual milk that was available for production. In the 2‐donor model, 65 (16.9%) donors were not selected for the simulation. These were predominantly low‐volume donors (average donation of 6.7 L vs 37.2 L for donors selected by the simulation; *p* < 0.0001). In the 10‐donor model, only 1 donor was not selected in the simulation, which is indicative of the greater number of opportunities to be selected when choosing 10 donors for each pool.

Lactose NII's differed by less than 5% between modeled and empirical values for 2‐donor, 3‐donor, 4‐donor, and 5‐donor pools (Figure 1C). Sodium NII's were more similar between empirical data and the Slow inventory turnover model (differences of −2.7% to 11.7%) than the Fast inventory turnover model (differences of 14.5%− 34.5%). A sensitivity analysis of model assumptions (Figure 1D) showed that inventory turnover did not influence NII values for nutrients that were less variable (e.g. lactose). For more variable nutrients like sodium, the Fast Inventory turnover produced higher NII's than the Slow Inventory turnover, with the models converging as more donors were added to the pool. There was a less than a 10% difference in NIIs between the Slow and Fast models for all nutrients at 4 or more donors per pool except sodium (< 10% difference achieved at 5‐donor pools); lactoferrin and iron (< 10% difference achieved at 6‐donor pools); and zinc (< 10% difference achieved at 7‐donor pools).

### 3.2 Impact of Multi‐Donor Pooling

**Figure 2:**
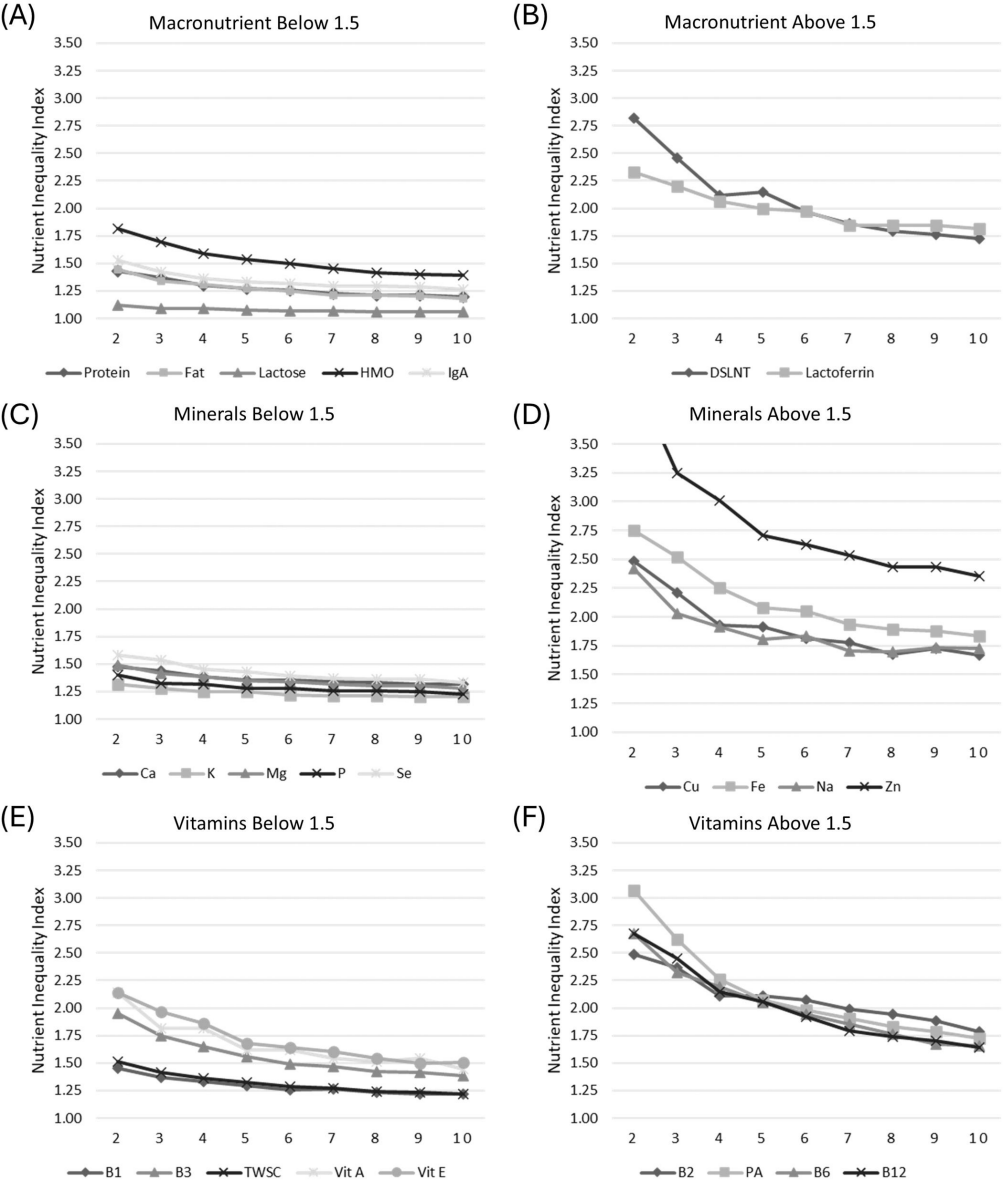
Nutrient Inequality Index (NII) by the number of donors per pool and nutrient class for macronutrients and bioactive proteins (A, B); minerals (C, D) and vitamins (E, F) grouped by nutrients with NII below and above 1.5. Note: Zinc NII value at 2‐donors (4.08) is not shown on graph D. Similarly, Biotin values declined from 40.4 to 15.7 and did not fit on the scale of graph F. B1 – thiamin; B2 – riboflavin; B3 – niacin; DSLNT ‐ disialyllacto‐N‐tetraose; HMO – human milk oligosaccharides; PA – pantothenic acid; TWSC – total water‐soluble choline.

Pooling results presented reflect the Slow Inventory turnover model, which more closely aligned with empirical results across 2–5 donors per pool. Nutrient Inequality Indices are presented in Figure 2, grouped by nutrient class (macronutrients, vitamins, and minerals) and whether the NII values were above or below 1.5. Lactose was the least variable nutrient at all pooling scenarios, with an NII ranging from 1.12 for 2‐donor pools to 1.06 for 10‐donor pools. True protein and fat visually stabilized around 5 or more donors per pool at an NII below 1.3 (Figure 2A). Lactoferrin and the human milk oligosaccharide DSLNT, which we considered as specific macronutrients, had more variability, with NII's around 1.75 at 10‐donor pools (Figure 2B). Potassium had the lowest mineral variability at all pooling scenarios, with an NII ranging from 1.32 for 2‐donor pools to 1.20 for 10‐donor pools (Figure 2C). Calcium, magnesium, phosphorus, and selenium had NIIs that stabilized below 1.5 at 5 or more donors per pool. Copper, sodium, iron, and zinc were more variable minerals (Figure 2D), with zinc being the most variable (2‐donor NII = 4.08 and 10‐donor NII = 2.43). Thiamin and total water‐soluble choline (Figure 2E) had the lowest variability for vitamins (2‐donor NII of 1.45 and 1.51 respectively; 10‐donor NII of 1.22 and 1.23 respectively). The most variable vitamin was biotin (2‐donor NII = 40.23 and 10‐donor NII = 15.73). Other vitamins with an NII above 1.5 for all pooling scenarios included riboflavin, pantothenic acid, B6, and B12 (Figure 2F).

### 3.3 Minimum Donors to Meet Clinical Targets

None of the random pooling scenarios we evaluated (2–10 donors) were able to ensure that at least 80% of the DHM pools (the 20th percentile) met the a priori nutrient targets of: true protein concentrations of at least 0.9 g/dL; total fat concentrations of at least 3.5 g/dL; and DSLNT concentrations of at least 210 µg/L (Table 2). At 5‐donors per pool, which is the minimum number of donors where an NII of < 1.3 was achieved for macronutrients, the 20th percentile for the target nutrients was: true protein of 0.73 g/dL; total fat of 2.9 g/dL; and DSLNT of 66 µg/L (Table 2). Less than 10% of the 5‐donor pools met a priori nutrient target for true protein ≥ 0.9 g/dL, 10% of the pools contained fat ≥ 3.5 g/dL, and 0% of the pools achieved a DSLNT concentration of 210 µg/L. At 10‐donors per pool, the percentage of pools meeting these targets was similar to the 5‐donor pools.

**Table 2:** 20th percentile values of the slow turnover simulations of 1000 pools of holder pasteurized donor milk created under 9 different pooling scenarios (2–10 randomly pooled donors).

# of Random donors per pool:	2	3	4	5	6	7	8	9	10
A priori nutrients:									
True protein (g/dL)	0.69	0.70	0.72	0.73	0.73	0.73	0.73	0.74	0.74
Fat (g/dL)	2.8	2.9	2.9	2.9	2.9	2.9	3.0	3.0	3.0
DSLNT (mg/L)	58	63	67	66	69	71	73	74	74
Secondary nutrients:									
Lactose (g/dL)	6.5	6.6	6.6	6.6	6.6	6.6	6.6	6.7	6.7
HMO Sum (mg/dL)	4018	4182	4323	4406	4412	4481	4466	4573	4574
Lactoferrin (mg/dL)	41	42	44	45	45	45	46	46	46
IgA (mg/dL)	168	175	178	178	179	180	182	180	181
NPN (%)	30	31	31	31	31	32	32	32	32
Thiamin/B1 (µg/L)	115	117	119	121	122	121	123	123	123
Riboflavin/B2 (µg/L)	201	212	223	231	232	238	238	240	246
Niacin/B3 (µg/L)	1089	1138	1174	1196	1220	1229	1228	1252	1255
Pyridoxine/B6 (µg/L)	107	114	117	121	122	123	127	129	128
B12 (pmol/L)	212	226	233	239	245	248	256	257	261
Biotin (µg/L)	5.8	6.4	7.1	7.8	9.3	11.1	11.8	12.5	13.8
Pantothenic acid (µg/L)	2517	2692	2842	2896	2988	3008	3068	3163	3173
Water‐soluble choline (mg/L)	79	82	83	84	85	85	86	86	86
α‐tocopherol (mg/L)	3.5	3.7	3.8	4.0	4.0	4.1	4.1	4.1	4.1
δ‐tocopherol (mg/L)	0.9	0.9	1.0	1.0	1.0	1.0	1.0	1.0	1.0
Retinol (mg/L)	0.3	0.4	0.4	0.4	0.4	0.4	0.4	0.4	0.4
Calcium (mg/L)	225	228	230	234	233	235	235	237	235
Copper (ppb)	155	165	173	176	180	182	184	183	187
Iron (ppb)	117	127	133	137	140	139	142	142	144
Magnesium (mg/L)	27.7	28.2	28.7	28.8	28.9	29.2	29.2	29.4	29.6
Phosphorus (mg/L)	119	122	122	124	124	124	124	124	125
Potassium (mg/L)	407	411	417	419	420	420	421	422	420
Selenium (ppb)	7.5	7.6	7.8	7.9	7.9	8.0	8.0	8.0	8.0
Sodium (mg/L)	73	77	79	80	81	82	82	83	83
Zinc (ppb)	862	930	1020	1070	1076	1110	1092	1122	1158

*Notes:* Data reflect the 20th percentile in the distribution of 1000 randomly simulated pools by the number of donors in the pool (2 to 10).

Abbreviations: DSLNT ‐ disialyllacto‐N‐tetraose; HMO – human milk oligosaccharides; IgA – immunoglobulin A; NPN – non‐protein nitrogen; WS – water‐soluble.

## 4 Discussion

In this simulation study that evaluated the impact of randomly pooling milk from 2 to 10 donors, we found that macronutrients were less variable than most vitamins and minerals at all pooling‐scenarios, and that a priori targets (80% of pools contained at least 0.9 g/dL of true protein, 3.5 g/dL of fat, and 210 µg/L of DSLNT) could not be achieved with any random pooling scenario. Use of a novel index, the Nutrient Inequality Index (NII), allowed for easy visual comparison of milk nutrients that are present across a range of concentrations (µg to g). While milk bank inventory turnover is unknown, our Slow turnover model had the best fit with empirical data and converged with the Fast turnover model at 4‐donors per pool for most nutrients, suggesting that multi‐donor pooling may also be a useful technique for smoothing DHM composition between milk banks that have different inventory turnover rates.

### 4.1 Impact of Pooling on Macronutrient Variability

Prior pooling studies have primarily evaluated the impact on DHM macronutrients with up to 5‐donors per pool. Two studies simulated the macronutrients in donor milk during random pooling of two to five donors using underlying macronutrient data from a single milk bank, assessed using infrared analysis (John et al. 2019; Tabasso et al. 2023). Both studies reported that the variability (maximum/minimum) in fat was greater (2.6 to 3.3‐fold) than the variability in protein (2.0‐fold in both studies) when randomly pooling 5‐donors. Our study reported the NII (ratio of the 90th to the 10th percentile) which is not directly comparable to maximum and minimum values reported in prior studies. At 5‐donors per pool, we observed that the fat and true protein NIIs were both 1.27, which is lower than the maximum/minimum ratio reported by John and Tabasso. Factors that may explain these differences include: using maximums and minimums reflects outlier values, while the NII limits the impact of outliers by assessing the core 80% of the pools; prior simulations did not weight the donor selection by lifetime donation volume, which may have under‐represented donors that contributed large volumes of milk. When fat and protein were directly measured in 300 samples collected systematically from 20 HMBANA milk banks, in the 5‐donor pools (*n* = 11) fat varied (maximum/minimum) by 1.3‐fold and protein varied by 1.4‐fold, which is closer to the NII values we observed in our simulated pools (Friend and Perrin 2020). Similarities should be interpreted with caution given that empirical results were based on a small number of samples. Our work extended simulation analysis to 10 donors per pool and found that improvements in NII tapered around five to six donors per pool, suggesting that this may be a sweet spot for milk banking to reduce macronutrient variability while also limiting the risk of contamination and recall with larger numbers of donors in a pool. Others have used targeted pooling in combination with machine‐learning to guide pooling decisions related to optimizing the protein and fat in donor human milk (Wong et al. 2021).

### 4.2 Impact of Pooling on Micronutrient Variability

Limited research has been done on how pooling impacts micronutrients in DHM. This is particularly important because micronutrient composition is not easily assessed in a milk bank setting, making random pooling one of the only tools currently available to address micronutrient variations in donations. In a study of pooled DHM from a single milk bank, variations in zinc were driven by the lactation stage of the DHM and were not improved in 3+ donor pools compared to smaller donor pools; however, there were only 11 pools containing 3+ donors which may not have provided sufficient power to detect differences (Young et al. 2019). The larger NIIs we reported for minerals like zinc, sodium, and copper may be explained by the fact that these nutrients decline by 50% or more in the early postpartum period (Allen et al. 2025), which may drive differences in DHM based on the individual lactation stages of the donations in the pool. Similarly, biotin rapidly increases in the early postpartum period (Allen et al. 2025) which likely contributed to the high NII values we observed for biotin. We did not estimate the blended lactation stages for each simulated pool. While drivers of variation in human milk micronutrient composition are not well understood, other factors include genetic difference (e.g. zinc transporters), maternal status, maternal diet, and sample collection protocol. To our knowledge, no prior studies have looked at the impact of donor pooling on vitamins in DHM. Several vitamins including biotin, riboflavin, pantothenic acid, and B12 had NIIs above 2.0 at 5‐donor pools, and above 1.5 at 10‐donor pools. Whether the wide variations we report in DHM micronutrients can meet preterm infant nutrient requirements when used with existing fortifier products is an important future area of research.

### 4.3 Meeting A Priori Clinical Targets

While milk banks can reduce nutrient variability with random pooling, our simulation indicated that the majority of DHM pools would not meet a priori targets for important nutrients in the NICU including 0.9 g/dL of true protein, 3.5 g/dL of fat, and 210 µg/L of DSNLT. Our findings related to protein are in contrast with other simulation studies on donor pooling. John et al. (2019) simulated the creation of 2000 random pools using donor information from a single milk bank and reported that at 5‐donors per pool, 90% of the pools would meet a true protein target of 1.0 g/dL. Tabasso et al. (2023) simulated 10,000 random pools using donor information from a single milk bank and reported that at 5‐donors per pool, 81% of the pools had protein concentrations > 0.9 g/dL. In contrast, we found that less than 10% of pools reached a protein target of 0.9 g/dL. A potential explanation for this difference is that we used true protein values assessed using a modified Kjeldahl technique, while the other studies used infrared analysis and were unclear if they were reporting crude protein values or true protein. Use of crude protein values would overstate protein concentrations (Belfort et al. 2024).

A biological explanation for the lower protein content that we observed in DHM is that the donations were predominantly collected at 3–4 months postpartum (Perrin et al. 2025) when protein concentrations in milk have been shown to be lower than the early postpartum or weaning periods. In the MILQ study of 1000 health lactating women followed longitudinally for 8.5 months, the median protein concentration at 3–4 months postpartum was 0.8 g/dL which is in agreement with our findings (Lewis et al. 2025). Given that some clinical reference values for DHM report protein concentrations of 1.2 g/dL (Infant and Pediatric Feedings 2019), emerging evidence suggests that revisions may be warranted.

We report that only 10% of 5‐donor pools would meet fat targets of 3.5 g/dL which is similar to those reported by Tabasso et al. (2023) where only 35% of the 5‐donor pools reached 3.5 g/dL of fat, and to a systematic review of DHM composition where 8/14 studies reporting average fat values below 3.5 g/dL (Perrin et al. 2020). In contrast, John et al. (2019) reported that 70% of 5‐donor pools would meet fat targets of 3.5 g/dL. Differences in simulation decisions may contribute to these differences related to fat. For instance, we used lifetime donation volume to determine how often a donor was selected for a pool, which may have generated lower fat values if high‐volume donors donated lower‐fat milk. Milk banks may be able to create higher fat pools using target pooling, where donors are selected for a pool based on their measured macronutrient composition (John et al. 2019), but it is possible this could also reduce DHM inventory if lower fat milk was no longer eligible to be selected for pools.

### 4.4 Strengths and Limitations

Strengths of this study include: the use of a large underlying database of 386 approved US milk bank donors; evaluation of macro‐ and micronutrients that have not previously been characterized as it relates to pooling; extension of pooling scenarios to 10‐donors per pool; and use of several techniques in our simulation to improve the trustworthiness of findings including weighting donor selection based on lifetime donation volumes, creating pools volumes reflective of milk banking practices, exploring the sensitivity of findings to model assumptions about inventory turnover rates, and comparing simulated NII values for two to five donors per pool against empirical findings. Study limitations include the absence of data on several essential micronutrients including folate, vitamin C, vitamin D, and vitamin K. We did not simulate beyond 10 donors to reflect the processing constraints within HMBANA milk banks, so these findings are not generalizable to commercial donor milk produced in much larger volumes or to DHM produced in non‐US settings or in settings where multi‐donor pooling is not culturally appropriate. The impact of pasteurization was considered at a nutrient level instead of at a milk sample level, which doesn't account for potential interdependence of nutrients within a milk sample; however, overall the loss after pasteurization was minimal to none for essential nutrients (Davis et al. 2025) which reduces the potential impact of this limitation. A larger number of donors were never selected by our simulation for the 2‐donor pools than for the 10‐donor pools, but overall, this was a relatively small percentage (< 0.1%–16.9%). We did not consider targeted pooling, where milk banks make strategic pooling decisions based on measured nutrient content of each donor, because many milk banks globally do not have access to macronutrient analyzers.

## 5 Conclusion

Random multi‐donor pooling of 2–10 donors produced lower variability in DHM macronutrients than the variability in most vitamins and minerals. Further, a priori targets (80% of pools containing at least 0.9 g/dL of true protein, 3.5 g/dL of fat, and 210 µg/L of DSLNT) could not be achieved with any random pooling scenario when donations occurred predominantly at 3–4 months postpartum. The NII for lactose stabilized at less than 1.1 when there were 3 or more donors per pool, while the NII for fat and true protein stabilized at less than 1.3 when there were 5 or more donors per pool. The NII exceeded 1.5, even at 10 donors per pool, for several micronutrients including zinc, copper, sodium, iron, biotin, riboflavin, B6, B12, and pantothenic acid. The extent that DHM meets preterm infant nutrient requirements is an important area of future research given the wide micronutrient variations reported in this study that persisted when randomly pooling 5 to 10 donors. Empirical research to confirm findings of pooling simulations is also warranted.

## Author Contributions

K.M., K.I.B., M.T.P., and S.R. conceptualized and designed the study. L.H.A., L.B., D.H., S.S.‐F., and M.T.P. contributed to sample analysis. R.M.S., E.I., S.R.,and M.T.P. contributed to simulation and statistical analysis. R.M.S., E.I., and M.T.P. drafted the manuscript. All authors reviewed and approved the manuscript.

## Conflicts of Interest

M.T.P. serves on the Scientific Advisory Board for the Human Milk Banking Association of North America, and on the World Health Organization Guidelines Development Group for Donor Milk Banking in unpaid capacities. K.I.B. and K.M. serve as Coordinators for the World Health Organization Standards on Human Milk Banking. L.B., L.H.A., D.H., E.I., S.R., S.S.‐F. and R.M.S. have no conflicts to report.

## Data Availability

The data that support the findings of this study are openly available in NICHD Data and Specimen Hub at https://dash.nichd.nih.gov/.
